# Age-Associated Reduction of Asymmetry in Human Central Auditory Function: A 1H-Magnetic Resonance Spectroscopy Study

**DOI:** 10.1155/2013/735290

**Published:** 2013-10-09

**Authors:** Xianming Chen, Yonghui Liang, Yihong Deng, Jianzhong Li, Shiyan Chen, Cuixia Wang, Ping Luo

**Affiliations:** ^1^Department of Otolaryngology-Head and Neck Surgery, Fuzhou General Hospital of Nanjing Command, PLA, 156 Xihuan Bei Road, Fuzhou 350025, China; ^2^Department of Otolaryngology-Head and Neck Surgery, Dongfang Hospital, Fujian Medical University, Fuzhou 350001, China

## Abstract

The aim of this study was to investigate the effects of age on hemispheric asymmetry in the auditory cortex after pure tone stimulation. Ten young and 8 older healthy volunteers took part in this study. Two-dimensional multivoxel 1H-magnetic resonance spectroscopy scans were performed before and after stimulation. The ratios of N-acetylaspartate (NAA), glutamate/glutamine (Glx), and **γ**-amino butyric acid (GABA) to creatine (Cr) were determined and compared between the two groups. The distribution of metabolites between the left and right auditory cortex was also determined. Before stimulation, left and right side NAA/Cr and right side GABA/Cr were significantly lower, whereas right side Glx/Cr was significantly higher in the older group compared with the young group. After stimulation, left and right side NAA/Cr and GABA/Cr were significantly lower, whereas left side Glx/Cr was significantly higher in the older group compared with the young group. There was obvious asymmetry in right side Glx/Cr and left side GABA/Cr after stimulation in young group, but not in older group. In summary, there is marked hemispheric asymmetry in auditory cortical metabolites following pure tone stimulation in young, but not older adults. This reduced asymmetry in older adults may at least in part underlie the speech perception difficulties/presbycusis experienced by aging adults.

## 1. Introduction

Hemispheric lateralization, also known as hemispheric asymmetry, refers to the structural or morphological differences between the right and left hemispheres, as well as the differences in the information processing capacity between the hemispheres [1]. For instance, dichotic listening test findings indicate that people hear verbal sounds, such as syllables or words, more precisely in the right ear than in the left ear. This is known as the right ear or left hemisphere advantage [2]. In contrast, people perceive nonverbal sounds, such as ambient noise and composite sound, more accurately in the left ear than in the right ear. This is known as the left ear or right hemisphere advantage [2]. The auditory cortex is an advanced center in the auditory system that is an important mediator of functional asymmetry, as confirmed by neuroanatomical, neuroimaging, and behavioral studies [3–9]. Morphological and metabolic changes in the auditory cortex may mediate the asymmetry of auditory center function, thereby affecting the analysis, integration, and perception of sound information. Factors that may affect the morphology and function of the auditory center, leading to remodeling and repeated remodeling, include aging, acquired learning (music and sign language), and hearing loss [10–16].

Age-related hearing loss, also known as presbycusis, is characterized by progressive aging of auditory organs and other tissues and organs which is accompanied by hearing impairment. Presbycusis cannot only decrease quality of life but may also cause abnormal psychological behavior or even lead to dementia, increasing the burden for families and society. The difference between presbycusis and other types of deafness is the reduced ability to distinguish speech. This may be related to functional remodeling of the auditory center, which in turn may be related to aging and reduced or absent peripheral input to the auditory center. The effects of aging on asymmetry of auditory center function remain controversial. The findings from a large number of early dichotic listening tests indicate that asymmetry of speech processing increases in elderly people, regardless of hearing loss, and that the main manifestation of this is the left ear disadvantage [11]. In contrast, another study, involving young and elderly subjects who were required to perform competitive verbal tasks, found that asymmetry of the left hemispheric advantage was decreased or even reversed in elderly subjects [12]. Clearly further research is needed to better understand the effects of aging on asymmetry of auditory center function. This may help provide a basis for the early diagnosis of presbycusis and hearing rehabilitation.

There is some evidence from animal histopathologic studies that functional asymmetry may be related to differences in biomarker content (including choline, nitrogen oxide synthase, and 17*β*-hydroxysteroid dehydrogenase) between bilateral cerebral hemispheres [6]. However, there have been very few studies on the metabolism of human brain bilateral auditory cortical neurons and neurotransmitters in different functional states due to a lack of noninvasive, objective, and accurate means for examining activity of the brain auditory cortex. 1H-magnetic resonance spectroscopy (1H-MRS) uses nuclear magnetic resonance and chemical shift to noninvasively and accurately detect energy metabolism and biochemical changes in living tissue. 1H-MRS can detect metabolite signals including N-acetyl-aspartate (NAA), choline-containing compounds (Cho), creatine compounds (Cr: creatine and phosphocreatine), lactic acid (Lac), myoinositol, *γ*-amino butyric acid (GABA), lipid, and glutamine/glutamate (Glx). Of these, NAA is related to nerve density and is considered a marker of neuronal damage, death, or metabolic inhibition, Cr is a marker of energy-dependent system of brain cells, and Glx and GABA are important excitatory and inhibitory neurotransmitter in the cerebral cortex [17].

In this study, we tested the hypothesis that asymmetry of the auditory cortex is associated with asymmetry in the content of cortical metabolites (NAA, Cr, Glx, and GABA) and that this asymmetry in metabolites is affected by age. Specifically, we used two-dimensional multivoxel 1H-MRS to examine bilateral auditory cortical metabolite changes in young and older subjects with normal hearing before and after pure tone stimulation. In order to eliminate the potential influences of confounding factors, such as cognitive recession and reduced peripheral input, we included older subjects with normal cognitive function and a pure tone hearing threshold within the normal range to observe the effects of simple aging on central auditory functional reorganization.

## 2. Materials and Methods

### 2.1. Subjects

Eighteen healthy volunteers took part in the study. There were 10 subjects (5 males and 5 females) with a mean age of 25.3 years (range: 18 to 27) in the young group, and 8 subjects (4 males and 4 females) with a mean age of 66.0 years (range: 55 to 75) in the older group. All subjects were righthanded. None of the subjects had any abnormalities in the external auditory canal or tympanic membrane as determined by otoscopy. The pure-tone threshold was 125~4000 Hz and the average hearing threshold was ≤25 dB. The pure-tone threshold was determined using a GSI 61 diagnostic pure tone audiometer (Grason-Stadler, Eden Prarie, MN, USA). Objective calibration and measurement were performed according to the guidelines provided by the manufacturer. All subjects had normal middle ear function as determined by tympanometry. No subjects had complaints of hearing loss or impairment, symptoms of vestibular system disturbance, such as tinnitus or dizziness, or had a history of neurological or psychiatric disorders, or other systemic diseases. 

Subjects with mild cognitive impairment, as determined using the Montreal Cognitive Assessment, were excluded (all subjects were required to score ≥26 points to be included in the study). Testing was performed by a neurologist with appropriate training using the Chinese version of the Montreal Cognitive Assessment.

This study was approved by the Ethics Committee of Dongfang Hospital. All subjects provided written informed consent before commencing any study-related procedures.

### 2.2. Study Design

**Figure 1:**
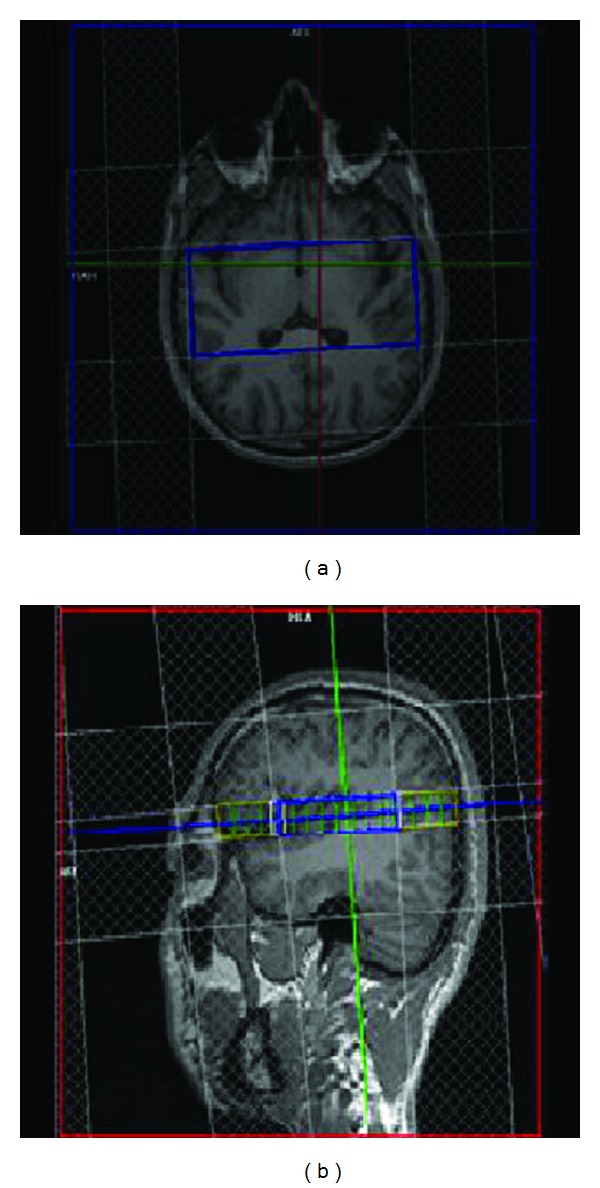
Representative two-dimensional multivoxel axial and sagittal positioning images. The central frame in the left image highlights the rectangular region of interest for data acquisition. The left image also illustrates that the acquisition region covers the bilateral auditory cortex. The right image demonstrates that the positioning image is parallel to the lateral fissure.

For each subject, the study was carried out by a physician experienced in MRS operation and diagnosis. Subjects were placed in a supine position on the MRI table and were required to wear goggles to eliminate visual stimulation. A conventional three-dimensional MRI scan was firstly performed to exclude intracranial lesions. Axial positioning was selected for two-dimensional multivoxel 1H-MRS of the bilateral acoustic cortex (Figure 1). The positioning image used the cross-section parallel to the lateral fissure. Each subject underwent an initial MRS scan in a relatively quiet state (spectra were collected). Five minutes later, a sine wave pure tone pulse stimulation with a sound intensity of 90 dB and a frequency of 1000 Hz was applied to both ears. The pulse stimulation continued for 500 ms at a frequency of 1 Hz. The sine wave pure tone was generated using Adobe Audition 2.0 software (Adobe system, CA, USA), and the stimulation program was prepared using E-prime software (Psychology software tools, Inc., Philadelphia, PA, USA) with output to a SAMRTEC SAV28800 magnetic resonance auditory stimulation device. The sound was applied to subjects' ears using magnetic resonance-compatible headphones. Subjects were asked to focus on listening to the sound from the headphones and maintain regular breathing. The second MRS scan was performed at the time of sound stimulation.

### 2.3. Magnetic Resonance Spectroscopy

A Siemens Trio Tim 3.0 T MRI scanner (Siemens Medical Solutions, Erlangen, Germany) and a 24-channel standard quadrature head coil were used for data acquisition. The parameters were RF-FAST T1-weighted three-dimensional images; TR/TE = 1700 ms/135 ms; 256 × 256 matrix; slice thickness = 1 mm; slice interval = 0 mm. The region of interest (ROI) included the planum temporal of the bilateral auditory cortex, the entire Heschl's gyrus, and part of the insular cortex. Calibration was performed by combining the coronal and sagittal observation so that the ROI conformed to the anatomical characteristics of the auditory cortex and skull interference was avoided (Figure 1). Point-resolved spectroscopy was used for spectrum acquisition: the field of view was 12.2 cm × 6.4 cm; the number of excitations was 1; and the size of each voxel was 1.0 cm × 0.8 cm × 0.8 cm. The ROI covered the bilateral acoustic cortex, and the imaging time was approximately 20 min. The receiver/transmitter gain adjustment, voxel shimming, water suppression, and fat suppression image scans were completed automatically. The shimming effect reached a full width at half maximum <19 Hz and a water suppression level >95%.

### 2.4. Postprocessing of Data

**Figure 2:**
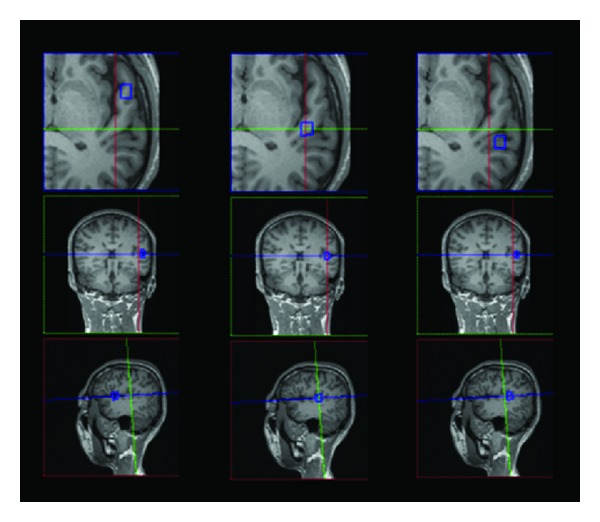
Representative images showing voxel selection from different regions of the left auditory cortex in the multivoxel spectra matrix for spectral reconstruction. The figure shows voxels selected from different regions of the anterolateral aspect of first Heschl's gyrus (left), the posteromedial aspect of first Heschl's gyrus (middle), and the planum temporal (right). Note: the middle blue frame indicates the region of interest. From top to bottom, the figure shows the axial, coronal, and sagittal positioning images.

The scanned csi-se-prostate sequence was uploaded to the Spectroscopy software for postprocessing. The metabolites observed by MRS included: NAA (located at 2.00 to 2.02 ppm, where ppm indicates the degree of the chemical shift), Cr (located at 3.02 to 3.03 ppm), Cho (located at 3.2 ppm), GABA (located at 2.28 to 2.31 ppm), Glx (located at 3.78 ppm), and Lac (located at 1.33 ppm). Cr was used as a reference to calculate the ratio of the area under the peak of NAA/Cr, Glx/Cr, and GABA/Cr. For each value, a corresponding voxel was selected from the bilateral anterolateral aspect of first Heschl's gyrus, the posteromedial aspect of first Heschl's gyrus, and the planum temporal in the multivoxel spectra matrix for the spectra reconstruction (Figure 2). The mean of the three regions was then calculated.

### 2.5. Statistical Analysis

Continuous variables are presented as median and interquartile range (the range between the 25th and 75th percentile) and were compared between age groups by Mann-Whitney *U* test. Differences between the left and right side within groups were compared by Wilcoxon signed rank test. Adjusting for before-stimulation variables, the after stimulation NAA/Cr, Glx/Cr, and GABA/Cr results were compared between two groups by analysis of covariance. SAS software package, version 9.2 (SAS Institute Inc., Cary, NC, USA), was used for statistical analyses. All statistical assessments were evaluated at a two-sided *α* level of 0.05.

## 3. Results

Table 1 summarizes the parameters of central auditory function for the young and older groups before and after pure tone stimulation. There were a number of significant between- and within-group differences detected.

Before stimulation, left and right side NAA/Cr and right side GABA/Cr were significantly lower, whereas right side Glx/Cr was significantly higher in the older group compared with the young group (all *P* ≤ 0.037). After stimulation, left and right side NAA/Cr and GABA/Cr were significantly lower, whereas left side Glx/Cr was significantly higher in the older group compared with the young group (all *P* ≤ 0.023). After adjusting for before-stimulation variables, after-stimulation left and right side NAA/Cr were significantly lower, whereas left side Glx/Cr was significantly higher in the older group compared with the young group (all *P* < 0.001). 

Before stimulation in the young group, right side GABA/Cr was significantly lower than left side GABA/Cr (*P* = 0.004). After stimulation in the young group, right side NAA/Cr and GABA/Cr were significantly lower than left side NAA/Cr and GABA/Cr, whereas right side Glx/Cr was significantly higher than left side Glx/Cr (all *P* ≤ 0.004). 

After stimulation in the older group, right side GABA/Cr was significantly lower than left side GABA/Cr, whereas right side Glx/Cr was significantly higher than left side Glx/Cr (both *P* ≤ 0.016). 

**Figure 3:**
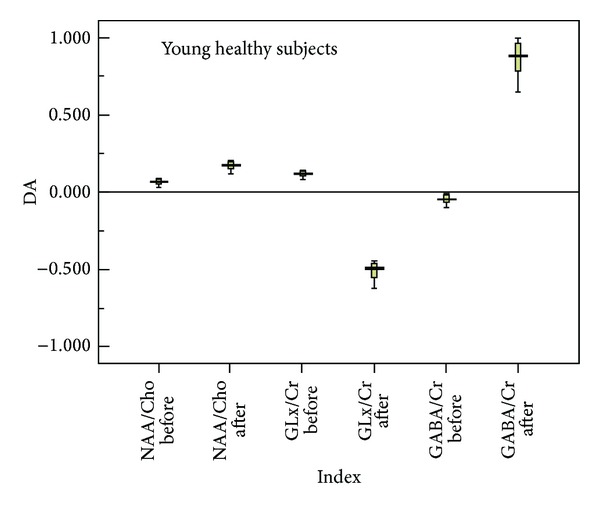
Schematic diagram of asymmetry of left and right auditory cortical metabolites in the young group before and after pure tone stimulation. Degree of asymmetry >0 indicates that the content of the auditory cortical metabolite on the left side is higher than that on the right side, whereas degree of asymmetry <0 indicates that the content of the auditory cortical metabolite on the right side is higher than that on the left side. Abbreviations: DA: degree of asymmetry; Cho: choline-containing compounds; Cr: creatine-containing compounds; Glx: glutamine/glutamate; NAA: N-acetyl-aspartate.

**Figure 4:**
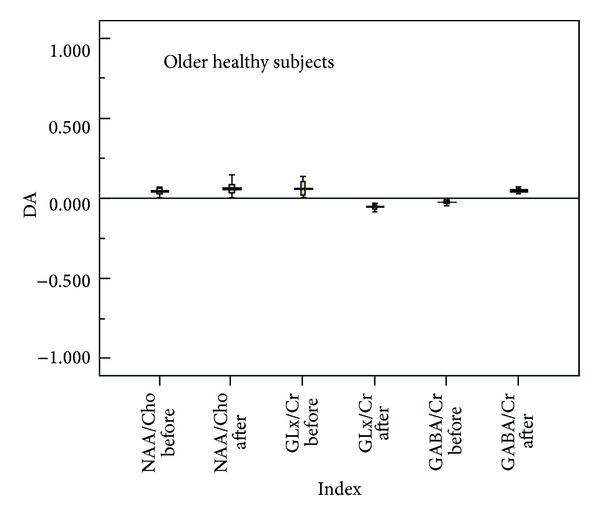
Schematic diagram of asymmetry of left and right auditory cortical metabolites in the older group before and after pure tone stimulation. Degree of asymmetry >0 indicates that the content of the auditory cortical metabolite on the left side is higher than that on the right side, whereas degree of asymmetry <0 indicates that the content of the auditory cortical metabolite on the right side is higher than that on the left side. Abbreviations: DA: degree of asymmetry; Cho: choline-containing compounds; Cr: creatine-containing compounds; Glx: glutamine/glutamate; NAA: N-acetyl-aspartate.

Figures 3 and 4 summarize the asymmetry of auditory cortical metabolites (NAA/Cho, Cho/Cr, and Glx/Cr), shown as the degree of asymmetry (left − right/left + right). Degree of asymmetry >0 indicates that the content of the auditory cortical metabolite on the left side is higher than that on the right side, whereas degree of asymmetry <0 indicates that the content of the auditory cortical metabolite on the right side is higher than that on the left side. In the young group after stimulation, there was marked right side asymmetry for Glx/Cr and left side asymmetry for GABA/Cr (Figure 3). In the older group, there was minimal asymmetry before and after stimulation (Figure 4).

## 4. Discussion

The findings from this study support our hypothesis that hemispheric asymmetry of the auditory cortex is associated with asymmetry in the content of cortical metabolites and that this asymmetry in the content of cortical metabolites is affected by age. Specifically, using two-dimensional multivoxel 1H-MRS, we found that there was clear right side asymmetry in Glx/Cr and left side asymmetry in GABA/Cr among subjects in the young group after pure tone stimulation. However, the extent of asymmetry was clearly less pronounced in the older group. These findings may have relevance to the pathology of age-related hearing loss/presbycusis.

In a previous study, Bellis et al. [18] assessed event-related potentials to examine asymmetry in the neurophysiological response elicited by compound syllables in different age groups and found that the amplitude of the P1-N1 evoked response in the auditory cortex detected in the left temporal lobe was greater than that on the right side in children and young adults, but that the response in elderly adults was symmetrical. The authors concluded that the asymmetry of verbal tasks was affected by aging, changing from the left side advantage to bilateral symmetry. Our finding that there was reduced bilateral asymmetry in the metabolite signals after pure tone stimulation in older subjects is consistent with the conclusion of Bellis et al. [18]. 

There are several possible explanations for the age-related change in the hemispheric asymmetry of sound processing by the auditory center. One explanation concerns changes in the brain structures. Indeed, Geal-Dor et al. [19] reported that atrophy of the temporal-parietal region of the brain occurs before that of other brain regions. Findings from other studies [18, 20] indicate that aging can cause progressive corpus callosum fiber atrophy and/or demyelination, which may further delay nerve conduction or reduce interhemispheric conduction efficiency. Another explanation is changes in cognitive function, which may be explained by two models: the right hemisphere-aging model (i.e., the right hemisphere shows more severe age-related dysfunction than the left hemisphere) or the hemispheric asymmetry reduction in old adults model (HAROLD) [21]. The latter is supported by findings from a large number of functional imaging studies related to cognitive function, episodic memory encoding, retrieval, semantic memory, working memory, cognition, and inhibitory control [18, 22]. For example, the activation of language working memory in the prefrontal lobe is left lateralized in young people, while the activation of spatial tasks is right lateralized. In contrast, both induce symmetrical activation in the elderly [23]. Kristofiková et al. [6] have suggested that the asymmetrical reduction in biomarkers with left/right laterality during the aging process in animals is consistent with the HAROLD model. The reduced asymmetrical distribution of metabolites in older subjects in the present study after pure tone stimulation is also consistent with the HAROLD model. 

Our finding that there was an interhemispheric difference in Glx/Cr after pure tone stimulation in the young group is consistent with the findings from several other comparable studies. For instance, in a blood oxygenation leve1-dependent functional magnetic resonance imaging and magnetoencephalography study, Geng et al. [24] found that the right hemisphere was the dominant hemisphere involved in auditory processing during pure tone stimulation. We have also previously reported that the auditory cortex shows right hemispheric dominance processing (as determined by functional imaging) after the same pure tone stimulation as used in the current study [25]. We suggest that the reduced excitability of the left auditory cortex might be related to competitive inhibition after activation of the right auditory cortex [26, 27]. 

Bishop and Miller [28] found that the auditory cortex on the left side can separate foreground sound from the background noise and thus proposed that various brain regions, including the medial temporal lobe in the left hemisphere, the left superior temporal sulcus, the parietal lobe, and the motor cortex, are involved in mediating the understanding of speech in noise through multisensory integration. In the present study, we found that GABA/Cr was significantly higher in the left auditory cortex after stimulation in both groups. This finding leads us to suggest that GABA may exert neuromodulatory effects and inhibit endogenous and exogenous noise, thereby contributing to improved hearing and speech discrimination. 

In the present study, we found that NAA/Cr in the auditory cortex in the older group was lower than that in the young group both before and after pure tone stimulation. This finding is consistent with that reported by Angelie et al. [29], who found, using two-dimensional 1H-MRS, that the NAA percentage decreased with age in the temporal lobe and semioval center of the human brain. The authors concluded that dysfunction caused by loss, damage, or plasticity/disappearance of neurons related to accelerated cell membrane transportation and changes in cellular energy metabolism are important neurophysiological mediators of brain aging.

Our study has several limitations that warrant mention. First, it is possible that age-related morphological changes in brain grey and white matter may have affected our results to some extent. Second, there may have been some variability in Cr content between different types of brain tissue that may have influenced our findings. However, Cr is considered to be the most stable brain metabolite [30] and therefore most appropriate for use as an internal standard in studies of this nature. Finally, our findings do not rule out the possibility that the observed changes are global hemispheric in nature rather than limited to the auditory cortex. Further studies are warranted to examine this possibility.

### 4.1. Conclusions

Multivoxel 1H-MRS allows for the assessment of metabolite content in different regions in the cerebral cortex following specific stimuli. In the present study, we found that there was asymmetry in the content of metabolites (Glx/Cr and GABA/Cr) with respect to hemispheric dominance for sound processing during pure tone stimulation in young adults. In contrast, there was clearly less-pronounced asymmetry detected in older adults, suggesting that the hemispheric dominance for pure tone processing may disappear with age. This reduced asymmetry in older adults may at least in part underlie the speech perception difficulties/presbycusis experienced by aging adults. This possibility warrants further investigation.

## Figures and Tables

**Table 1:** Comparison of central auditory function between the young and older groups before and after pure tone stimulation.

Variable	Young (*N* = 10)	Older (*N* = 8)	*P* value
Left-NAA/Cr			
Before	2.95 (2.75, 2.97)	1.90 (1.90, 1.96)	<0.001*
After	3.02 (2.96, 3.05)	1.80 (1.64, 1.98)	<0.001*
Adjusted difference^a^	reference	−1.189 (−1.730, −0.648)	<0.001*
Right-NAA/Cr			
Before	2.78 (2.75, 2.92)	1.88 (1.83, 1.94)	<0.001*
After	2.87 (2.85, 2.92)^†^	1.98 (1.89, 2.01)	<0.001*
Adjusted difference^a^	reference	−1.109 (−1.518, −0.699)	<0.001*
Left-Glx/Cr			
Before	0.12 (0.11, 0.13)	0.11 (0.10, 0.12)	0.657
After	0.06 (0.05, 0.07)	0.10 (0.09, 0.11)	<0.001*
Adjusted difference^a^	reference	0.040 (0.030, 0.051)	<0.001*
Right-Glx/Cr			
Before	0.09 (0.08, 0.10)	0.11 (0.11, 0.12)	0.037*
After	0.11 (0.10, 0.35)^†^	0.12 (0.12, 0.14)^†^	0.168
Adjusted difference^a^	reference	−0.047 (−0.141, 0.048)	0.311
Left-GABA/Cr			
Before	0.12 (0.11, 0.14)	0.07 (0.06, 0.33)	0.083
After	0.14 (0.13, 0.16)	0.08 (0.07, 0.08)	0.007*
Adjusted difference^a^	reference	−0.067 (−0.171, 0.037)	0.187
Right-GABA/Cr			
Before	0.10 (0.09, 0.11)†	0.07 (0.06, 0.08)	0.001*
After	0.07 (0.06, 0.07)^†^	0.05 (0.05, 0.06)^†^	0.023*
Adjusted difference^a^	reference	−0.010 (−0.023, 0.003)	0.126

Data are presented as median (interquartile range, the range between the 25th and 75th percentile) and were compared between groups by Mann-Whitney *U* test. Data were compared within groups between left and right by Wilcoxon signed rank test used.

Abbreviations: Cr: creatine-containing compounds; GABA: *γ*-amino butyric acid; Glx: glutamine/glutamate; NAA: N-acetyl-aspartate.

^a^Compared with the young group, the adjusted difference of older group is presented as difference and 95% CI. After Adjusting for before-stimulation variables, after-stimulation variables were compared between groups by analysis of covariance.

*Indicates a significant between-group difference (*P* < 0.05).

^†^Indicates a significant within-group difference between left and right (*P* < 0.05).

## References

[1] Toga AW, Thompson PM (2003). Mapping brain asymmetry. *Nature Reviews Neuroscience*.

[2] Schonwiesner M, Rübsamen R, von Cramon DY (2005). Hemispheric asymmetry for spectral and temporal processing in the human antero-lateral auditory belt cortex. *European Journal of Neuroscience*.

[3] Anderson B, Southern BD, Powers RE (1999). Anatomic asymmetries of the posterior superior temporal lobes: a postmortem study. *Neuropsychiatry, Neuropsychology and Behavioral Neurology*.

[4] Binder J, Frost JA, Hammeke TA (2000). Human temporal lobe activation by speech and nonspeech sounds. *Cerebral Cortex*.

[5] Galuske RAW, Schlote W, Bratzke H, Singer W (2000). Interhemispheric asymmetries of the modular structure in human temporal cortex. *Science*.

[6] Kristofiková Z, Rícný J, Ort M, Rípová D (2010). Aging and lateralization of the rat brain on a biochemical level. *Neurochemical Research*.

[7] Penhune VB, Zatorre RJ, MacDonald JD, Evans AC (1996). Interhemispheric anatomical differences in human primary auditory cortex: probabilistic mapping and volume measurement from magnetic resonance scans. *Cerebral Cortex*.

[8] Price CJ (2000). The anatomy of language: contributions from functional neuroimaging. *Journal of Anatomy*.

[9] Scott SK, Catrin Blank C, Rosen S, Wise RJS (2000). Identification of a pathway for intelligible speech in the left temporal lobe. *Brain*.

[10] Bilecen D, Seifritz E, Radü EW (2000). Cortical reorganization after acute unilateral hearing loss traced by fMRI. *Neurology*.

[11] Gonçales AS, Cury MCL (2011). Assessment of two central auditory tests in elderly patients without hearing complaints. *Brazilian Journal of Otorhinolaryngology*.

[12] Greenwald RR, Jerger J (2001). Aging affects hemispheric asymmetry on a competing speech task. *The Journal of the American Academy of Audiology*.

[13] Jerger J, Alford B, Lew H, Rivera V, Chmiel R (1995). Dichotic listening, event-related potentials, and interhemispheric transfer in the elderly. *Ear and Hearing*.

[14] Jerger J, Moncrieff D, Greenwald R, Wambacq I, Seipel A (2000). Effect of age on interaural asymmetry of event-related potentials in a dichotic listening task. *The Journal of the American Academy of Audiology*.

[15] Langers DRM, van Dijk P, Backes WH (2005). Lateralization, connectivity and plasticity in the human central auditory system. *NeuroImage*.

[16] Ohnishi T, Matsuda H, Asada T (2001). Functional anatomy of musical perception in musicians. *Cerebral Cortex*.

[17] Rosen Y, Lenkinski RE (2007). Recent advances in magnetic resonance neurospectroscopy. *Neurotherapeutics*.

[18] Bellis TJ, Nicol T, Kraus N (2000). Aging affects hemispheric asymmetry in the neural representation of speech sounds. *The Journal of Neuroscience*.

[19] Geal-Dor M, Goldstein A, Kamenir Y, Babkoff H (2006). The effect of aging on event-related potentials and behavioral responses: comparison of tonal, phonologic and semantic targets. *Clinical Neurophysiology*.

[20] Jerger J, Lew HL (2004). Principles and clinical applications of auditory evoked potentials in the geriatric population. *Physical Medicine and Rehabilitation Clinics of North America*.

[21] Dolcos F, Rice HJ, Cabeza R (2002). Hemispheric asymmetry and aging: right hemisphere decline or asymmetry reduction. *Neuroscience and Biobehavioral Reviews*.

[22] Logan JM, Sanders AL, Snyder AZ, Morris JC, Buckner RL (2002). Under-recruitment and nonselective recruitment: dissociable neural mechanisms associated with aging. *Neuron*.

[23] Reuter-Lorenz PA, Jonides J, Smith EE (2000). Age differences in the frontal lateralization of verbal and spatial working memory revealed by PET. *Journal of Cognitive Neuroscience*.

[24] Geng ZJ, Zhang YT, Zhang Q, Sun JL, Li SM (2006). Predominant hemisphere at pure tone stimulus: a study of fMRI combined with magnetoencephalography. *Journal of Clinical Radiology*.

[25] Mao CL, Chen XM, Chen ZQ, Ye YQ, Luo P (2009). fMRI study of auditory cortex in healthy subjects. *Chinese Journal of Practical Nervous Diseases*.

[26] Chiarello C, Maxfield L (1996). Varieties of interhemispheric inhibition, or how to keep a good hemisphere down. *Brain and Cognition*.

[27] Cook ND (1984). Callosal inhibition: the key to the brain code. *Behavioral Science*.

[28] Bishop CW, Miller LM (2009). A multisensory cortical network for understanding speech in noise. *Journal of Cognitive Neuroscience*.

[29] Angelie E, Bonmartin A, Boudraa A, Gonnaud P-M, Mallet J-J, Sappey-Marinier D (2001). Regional differences and metabolic changes in normal aging of the human brain: proton MR spectroscopic imaging study. *American Journal of Neuroradiology*.

[30] Brandao LA, Domingues RC (2003). *MR Spectroscopy of the Brain*.

